# Development of a Topical Amphotericin B and *Bursera graveolens* Essential Oil-Loaded Gel for the Treatment of Dermal Candidiasis

**DOI:** 10.3390/ph14101033

**Published:** 2021-10-12

**Authors:** Lupe Carolina Espinoza, Lilian Sosa, Paulo C. Granda, Nuria Bozal, Natalia Díaz-Garrido, Brenda Chulca-Torres, Ana Cristina Calpena

**Affiliations:** 1 Departamento de Química, Universidad Técnica Particular de Loja, Loja 1101608, Ecuador; lcespinoza@utpl.edu.ec (L.C.E.); bachulca@utpl.edu.ec (B.C.-T.); 2 Institute of Nanoscience and Nanotechnology (IN2UB), University of Barcelona, 08028 Barcelona, Spain; paulogranda92@gmail.com; 3 Faculty of Chemical Sciences and Pharmacy, National Autonomous University of Honduras (UNAH), Tegucigalpa 11101, Honduras; lilian.sosa@unah.edu.hn; 4 Department of Pharmacy, Pharmaceutical Technology and Physical Chemistry, Faculty of Pharmacy and Food Sciences, University of Barcelona, 08028 Barcelona, Spain; 5 Department of Biology, Healthcare and the Environment, School of Pharmacy and Food Sciences, University of Barcelona, 08028 Barcelona, Spain; nuriabozaldefebrer@ub.edu; 6 Department of Biochemistry and Physiology, Faculty of Pharmacy and Food Sciences, University of Barcelona, 08028 Barcelona, Spain; natalia.diaz.garrido@gmail.com; 7 Institute of Biomedicine of the University of Barcelona-Sant Joan de Déu Research Institute (IBUB-IRSJD), 08028 Barcelona, Spain

**Keywords:** amphotericin B, candidiasis, gel, essential oil, *Bursera graveolens*, topical treatment

## Abstract

The higher molecular weight and low solubility of amphotericin B (AmB) hinders its topical administration. The aim of this study was to incorporate *Bursera graveolens* essential oil into an AmB topical gel (AmB + BGEO gel) in order to promote the diffusion of the drug through the skin in the treatment of cutaneous candidiasis. AmB + BGEO gel formulation was determined using a factorial experiment. Physical and chemical parameters, stability, in vitro release profile and ex vivo permeation in human skin were evaluated. In vitro antimicrobial activity was studied using strains of *C. albicans*, *C. glabrata* and *C. parapsilosis*. The tolerability was evaluated using in vitro and in vivo models. AmB + BGEO gel presented appropriate characteristics for topical administration, including pH of 5.85, pseudoplastic behavior, optimal extensibility, as well as high stability and acceptable tolerability. In vitro release studies showed that the formulation releases the drug following a Boltzmann sigmoidal model. Finally, AmB + BGEO gel exhibited higher amount of drug retained inside the skin and lower Minimum Inhibitory Concentration than a formulation sans essential oil. Therefore, these results suggest that the incorporation of *B. graveolens* essential oil in the formulation could be used as strategy to promote a local effect in the treatment of cutaneous candidiasis.

## 1. Introduction

Cutaneous candidiasis is a superficial mycosis caused by proliferation in the skin by fungal organisms belonging to the genus *Candida* [1]. *C. albicans* is responsible for approximately 70% of skin infections associated with *Candida* spp. [2,3]. This type of mycosis mainly affects intertriginous areas and produces dryness, erythema, edema, erosions and pustules [4]. Topical treatments for cutaneous candidiasis include the application of azoles and polyenes such as clotrimazole, miconazole, fluconazole, itraconazole and nystatin [5,6,7]. However, resistance mechanisms both intrinsic and acquired to these antifungal drugs have been reported. Intrinsic resistance is highly stable and is predictive of therapeutic failure whereas acquired resistance can be either stable or transient and is developed due to long exposure to antifungals [8,9,10]. Although polyene resistance is still uncommon compared to resistance to other antifungals, some mechanisms of resistance have been proposed, including: (1) alterations in the sterol composition of the fungal cell membrane due to several mutations in genes (ERG) of the ergosterol biosynthesis pathway, (2) oxidative stress, and (3) alterations of the fungal cell wall characterized by an increased glucan production [11].

AmB is a polyene macrolide antibiotic produced by *Streptomyces nodosus* that acts by binding with the sterols (ergosterol) present in the cell membrane of susceptible fungi, resulting in the creation of transmembrane channels that induce ion permeability, loss of protons and monovalent cations, and consequently depolarization and concentration-dependent cell killing [12,13]. AmB has been clinically used to treat *Candida*, *Cryptoccocus* and *Aspergillus* via intravenous infusion [14,15]. However, its systemic adverse effects are well-known, including symptoms from acute toxicity such as nausea, vomiting, rigors, fever, hypertension or hypotension, and hypoxia as well as chronic adverse effects such as nephrotoxicity [16]. A topically administered AmB formulation can bypass these disadvantages from invasive administration techniques and thus permit treatment of localized infections in skin or mucous membranes [17]. However, the higher molecular weight (926 Da) and low solubility in water of AmB could limit its passage through the skin [18]. These complications could be circumvented by incorporating excipients with permeation-enhancing properties that promote the penetration of drugs through the stratum corneum (SC) and its distribution to the epidermis and dermis [19].

Examples of commonly studied permeation enhancers for dermal drug delivery include azone, pyrrolidones, sulphoxides, fatty acids, and surfactants as well as essential oils and their components [20]. Ideally, these compounds induce a temporary and reversible reduction in the barrier function of the SC in order to facilitate the drug permeation across the skin [21,22].

Essential oils are natural products extracted from aromatic plant materials and contain complex mixtures of several volatile compounds such as terpenes, terpenoids and phenylpropanoids [22]. Essential oils are widely used in pharmaceutical, cosmetic, agricultural and food industries in several applications including improvement of drug permeation in addition to antibacterial, antifungal, antioxidant and anti-inflammatory activities [23]. As permeation enhancers, the active compounds derived from essential oils increase drug diffusion by changes in the SC structure and interaction with intercellular lipids in the SC [22,23]. In particular, the *Bursera graveolens* essential oil has been reported to inhibit the growth of breast tumor cells and amastigotes of *Leishmania amazonensis* while also having antioxidant, antifungal and anticarcinogenic properties [24,25]. *B. graveolens* is a deciduous tree found from western Mexico to northwestern Peru. Its wood exhibits a characteristic sweet, spicy and balsamic aroma that has traditionally been used as incense [26]. It is widely referred to as “palo santo” by local populations, which in Spanish means “holy wood” [27]. Essential oil obtained from the fruit of *B. graveolens* primarily contains d-limonene (49.89%), α-phellandrene (37.64%) and menthofuran (6.08%) [25,27].

The purpose of this study was to develop and characterize a topical gel of AmB and *B. graveolens* essential oil (AmB + BGEO gel) as strategy to promote drug permeation through the SC and its retention in human skin in order to achieve a local effect in the treatment of cutaneous candidiasis.

## 2. Results

### 2.1. Solubility Studies

**Figure 1:**
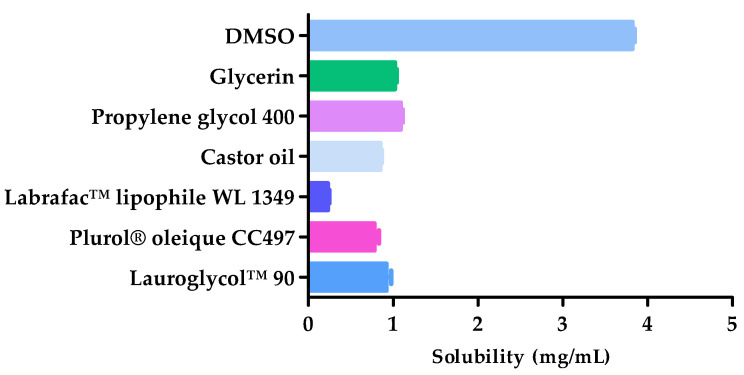
Amphotericin B (AmB) solubility in different excipients (*n* = 3).

The AmB solubility in different components are shown in Figure 1. DMSO (solubility 3.82 ± 0.05 mg/mL) exhibited greater ability to solubilize the drug and was, therefore, used as solvent in the formulation. Propylene glycol 400 (solubility 1.09 ± 0.04 mg/mL) and glycerin (solubility 1.02 ± 0.04 mg/mL) were evaluated by a 2^3^ factorial experiment in order to determine the most appropriate cosolvent for the formulation.

### 2.2. Design and Preparation of AmB + BGEO Gel Formulation

Table 1 shows the physical and chemical characterization of the eight formulations obtained by 2^3^ factorial experiment. After 60 days of storage at 30 °C, formulations 1, 4, 6 and 8 showed precipitates or formed lumps, whereas formulations 2 and 5 were homogeneous but showed significant changes in pH. These signs of instability were accompanied by a decrease in drug content. Finally, when comparing formulations 3 and 7, it was observed that formulation 3 was more stable, especially with regard to drug content, and was therefore selected as the final formulation for the AmB + BGEO gel.

AmB + BGEO gel (0.1%) was prepared by dissolving AmB in 5% DMSO, 20% Propylene glycol 400, and 2% *B. graveolens* essential oil. This mixture was then incorporated into a gel base composed of 3% CMC, 0.20% citric acid, 0.02% sodium benzoate, and 69.68% water (Table 2).

### 2.3. Characterization of AmB + BGEO Gel

AmB + BGEO gel showed a homogeneous appearance without signs of lumps or precipitates while also exhibiting a slightly yellowish color and a pleasant citrus scent characteristic of essential oil. The drug content in the formulation was of 99.65 ± 0.08% and with a pH value of 5.85 ± 0.09, which is biocompatible with the natural acidity of the skin.

**Figure 2:**
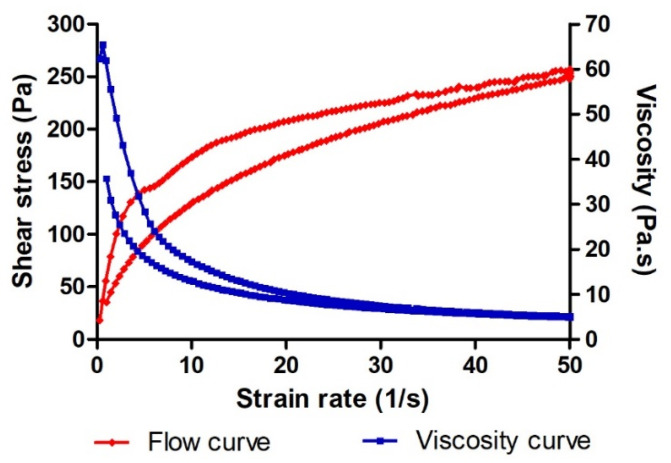
Rheological behavior of AmB + BGEO gel showing a hysteresis loop.

Rheological studies (Figure 2) confirmed pseudoplastic behavior of the formulation (Cross model r^2^ = 0.999) with a mean viscosity at 50 s^−1^ of 5036 ± 45.71 mPa·s. A hysteresis loop with an area of 4269 Pa/s was observed.

**Figure 3:**
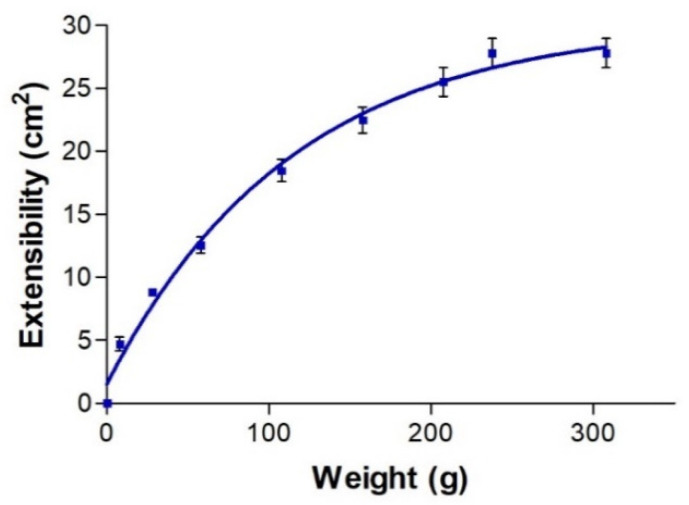
Extensibility profile of AmB + BGEO gel at 25 °C.

The extensibility profile of AmB + BGEO gel at 25 °C described in Figure 3 showed that the extensibility values increased proportionally to loading weight until reaching a maximum extensibility of 27.81 cm^2^ following a one-phase association mathematical model.

### 2.4. Stability Studies

The short-term stability evaluation of AmB + BGEO gel (Table 3) confirmed that after 120 days of storage at 30 °C and 40 °C, the formulation maintained a homogeneous aspect and suitable pH values for its application on skin (5.48 ± 0.12 to 5.85 ± 0.09 and 5.12 ± 0.07 to 5.83 ± 0.07, respectively). In the same way, the content of AmB present in the formulation remained without significant changes during the time of the stability study with a decrease of 0.86% at 30 °C and 1.1% at 40 °C.

### 2.5. In Vitro Release Study

**Figure 4:**
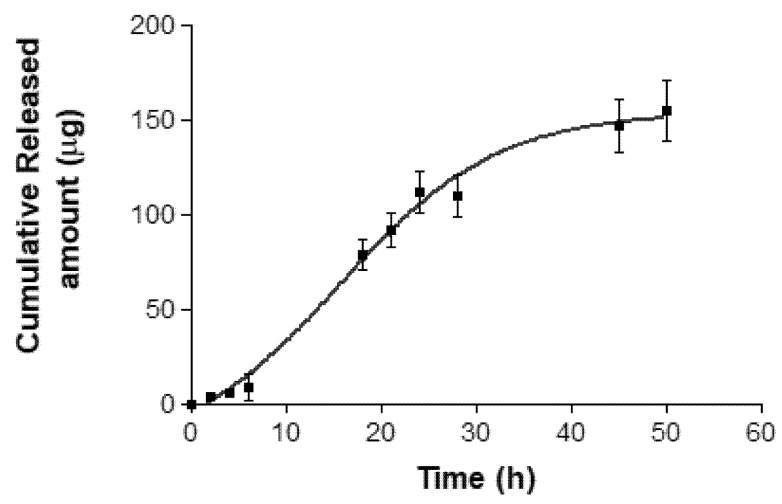
In vitro release profile of AmB from formulation. The cumulative amount released was plotted against time. Data represents mean ± SD (*n* = 3).

Figure 4 shows the release profile of AmB from the formulation. At the end of the experiment, an amount of 154.8 µg of AmB was released from the gel, which represents about 80% of the drug. The best fit of the experimental data was obtained with the Boltzmann sigmoidal model with a r^2^ = 0.9974 and whose mathematical equation is:

(1) $$Y=\mathrm{Bottom}+\left(\mathrm{Top}-\mathrm{Bottom}\right)/1+\exp\left(\frac{V50-x}{\mathrm{Slope}}\right)\;$$

where Y is the amount of released drug, Top and Bottom are the initial and final values of drug release, V50 is the time it takes to release half of the maximum amount susceptible to release and the Slope of the curve indicates the steepness.

### 2.6. Ex Vivo Permeation Study

Ex vivo permeation studies of AmB + BGEO gel and AmB gel (*B. graveolens* essential oil-free AmB gel) were carried out in order to compare and evaluate the role of the *B. graveolens* essential oil in drug permeation. AmB was not detected in the aliquots extracted from the receptor compartment over a 24 h period of permeation assay in either of the two formulations. However, AmB was found inside the skin with a retention value of 997.78 µg/g skin/cm^2^ for AmB + BGEO gel and 603.91 µg/g skin/cm^2^ for AmB gel.

### 2.7. Efficacy Study: Antimicrobial Activity

The antimicrobial activity after 48 h is reported in Table 4 as Minimum Inhibitory Concentration (MIC) values against strains of *C. albicans*, *C. glabrata* and *C. parapsilosis*. The *B. graveolens* essential oil at concentrations of 6.25 to 12.50% (*v*/*v*) showed antimicrobial activity against the studied *Candida* strains. The AmB + BGEO gel exhibited the lowest MIC values (0.29, 0.39 and 0.58 µg/mL) compared with AmB gel (0.58, 0.58 and 1 µg/mL) against *C. albicans*, *C. glabrata* and *C. parapsilosis*, respectively.

### 2.8. Tolerance Studies

#### 2.8.1. Cytotoxicity Studies by MTT Assay

**Figure 5:**
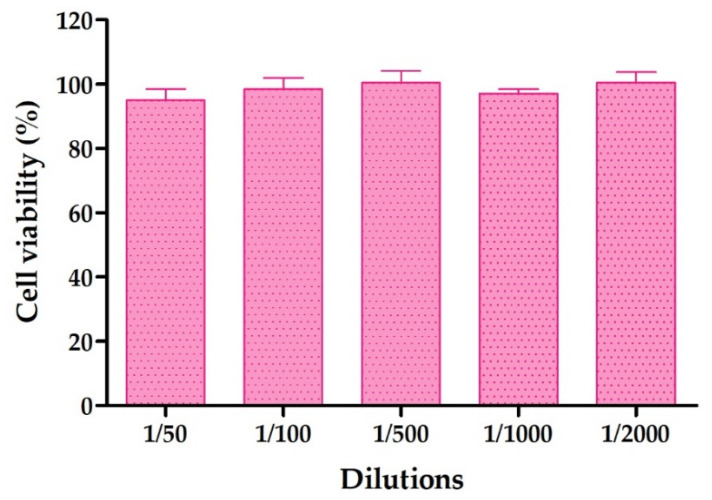
Percentage of cell viability of HaCaT cell line exposed to AmB + BGEO for 24 h at different concentrations.

The effect of different concentrations of AmB + BGEO gel on human keratinocytes was evaluated using MTT cytotoxicity assay. After 24 h of incubation, it was observed that the assayed dilutions of the formulation (1/50 to 1/2000) did not affect cell viability, which was close to 100% in relation to the control (Figure 5). Therefore, these results suggest that AmB + BGEO gel do not trigger toxicity in the cells.

#### 2.8.2. In Vivo Tolerance Studies

**Figure 6:**
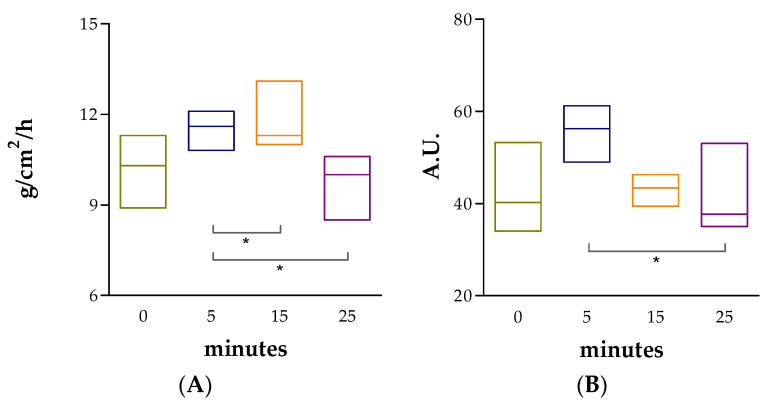
Tolerance studies in human individuals. (**A**) TEWL: Transepidermal water loss; (**B**) SCH: Stratum corneum hydration. Results are expressed as median, minimum and maximum (*n* = 12). Statistically significant differences: * *p* < 0.05.

No significant changes in the TEWL or SCH values with respect to the basal state were observed after 25 min of topical application of AmB + BGEO gel in the ventral area of the forearm of the volunteers (Figure 6). These results suggest that the formulation does not cause damage or irritation in the skin barrier.

## 3. Discussion

AmB is administered by intravenous infusion in the treatment of fungal infections [28]. This treatment causes several adverse effects including nausea, vomiting, rigors, fever, hypertension or hypotension, hypoxia and nephrotoxicity [16]. The incorporation of AmB into topical formulations could be used as a strategy to achieve a local effect in the skin without adverse systemic effects in the treatment of dermal candidiasis. However, its low aqueous solubility, great molecular weight (926 Da) and limited permeability of the SC to external substances, including drugs, could obstruct its penetration through the skin. In this study, the incorporation of *B. graveolens* essential oil into an AmB topical gel was carried out in order to promote drug permeation through the SC and its retention in human skin. The final formulation of AmB + BGEO gel (0.1%) was prepared using pharmaceutical excipients compatible with the drug (Table 2). This formulation was homogeneous with a pleasant citrus scent and had a slightly acidic pH value which assures non-irritating effects [29]. Determination of the flow properties of formulations, particularly those intended for skin application, provides important information about stability, sensory characteristics, spreadability and filling/dosing behavior [30]. A topical formulation should exhibit optimal viscosity and spreadability since a product too fluid or too viscous could be unpleasant or uncomfortable for patients [31,32]. Moreover, the viscosity and rheological behavior can modulate biopharmaceutical parameters such as release rates of drugs from their vehicles [33]. AmB + BGEO gel showed a mean viscosity of 5036 ± 45.71 mPa·s at 50 s^−1^ and a maximum extensibility of 27.81 cm^2^, which suggest that this formulation is easy to apply (Figure 2 and Figure 3). Rheological analysis and mathematical modeling confirmed the pseudoplastic behavior of AmB + BGEO gel and the presence of a hysteresis loop in the rheograms, suggesting thixotropic behavior [34]. These characteristics offer advantages for topical formulations since the viscosity decreases with friction, allowing for easy spreadability while returning to the initial state once the friction stops, which favors the residence in the treated area [35].

The stability studies at 30 and 40 °C for 120 days showed that AmB + BGEO gel maintained its homogeneous appearance and no significant changes in its pH values were detected after storage, showing values within the suitable range for skin application. Chemical stability was also observed with slight yet inconsequential changes in the quantified drug content, thus demonstrating high compatibility between the drug and the excipients (Table 3).

Interactions between the vehicle and drug define the release profile, which in turn affects the permeation rate of the drug [29]. In this research, the in vitro release study showed a released amount of 154.8 µg of AmB from the gel (Figure 4), which indicates that the vehicle is capable of releasing the drug without limiting skin permeation. According to the obtained r^2^ value, the release kinetic of AmB from gel followed a Boltzmann sigmoidal model that is characterized by an initial phase of slow-or-negligible release (0–6 h) followed by a second phase of immediate-or-controlled release [36].

Ex vivo permeation studies are used to estimate the in vivo behavior of the formulation, since the composition and the physical properties of the carrier influence the amount of drug reaching the target area [30]. The success of topical treatment is contingent upon the ability of the drug to cross the SC as well as its distribution through the epidermis and dermis [37,38]. The results of this study showed that the drug does not reach the receptor compartment in either of the two formulations (AmB + BGEO gel and AmB gel) but is instead retained in the tissue, thereby promoting a local effect in the skin without adverse effects. However, a higher amount of drug retained inside the skin was observed in the AmB + BGEO gel (997.78 µg/g skin/cm^2^) compared to AmB gel (603.91 µg/g skin/cm^2^), suggesting that the incorporation of *B. graveolens* essential oil facilitated the penetration of the drug through the SC and its diffusion to the epidermis and dermis. This essential oil contains limonene, a lipophilic terpene reported to have a skin permeation-enhancing effect of both hydrophilic and lipophilic drugs [39]. The proposed mechanism of this effect is due to changes in the intercellular packing and disruption in lipid structures that involve the modification of SC from solvents, thus improving drug partitioning into the skin [40,41].

The efficacy study showed that AmB + BGEO gel exhibited a lower MIC value against strains of *C. albicans*, *C. glabrata* and *C. parapsilosis* and is consequently more effective than the formulation sans essential oil (AmB gel). Significant antimicrobial activity of *B. graveolens* essential oil against strains of *C. albicans* has been previously reported. This effect has been attributed to its terpene-rich composition, namely limonene, although the exact mechanism is not completely understood as of yet. Nevertheless, some studies support the idea that these compounds could cause membrane disruption which consequently induces the death of the fungus [42]. A number of studies have shown that natural secondary metabolites with low molecular weight (≤500 g/mol) may act as adjuvants for antimicrobial drugs and thus potentiate its efficacy. Based on this approach, studies of combination therapy of antimicrobial drugs with terpenes have revealed promising effects against both susceptible and resistant pathogens [43]. In particular, essential oils such as *Citrus aurantium*, *Thymus kotschanus*, *myrtus communis* and *Mentha piperita* have shown a synergistic effect with clinical drugs including AmB, fluconazole, ketoconazole and meropenem [43,44,45,46]. In the current study, the combination of AmB with *B. graveolens* essential oil in a topical gel showed enhanced antifungal activity against *C. albicans*, *C. glabrata* and *C. parapsilosis.* These results indicate that the addition of *B. graveolens* essential oil in the formulation likely enhances the antimicrobial effect either due to its active compounds or a possible synergistic effect between its compounds and the drug.

The tolerability of AmB + BGEO gel was analyzed by in vitro and in vivo models. In vitro models are considered helpful to screen the toxicity of new formulations prior to the pre-clinical and clinical assessment. A variety of cell lines are frequently used for toxicity screening in a living system due to the cells being generally easy to cultivate, fast to grow and are sensitive to toxic irritation [47]. In this study, the tested dilutions of AmB + BGEO gel did not induce relevant cytotoxic effects on cells after 24 h of incubation, which confirms high biocompatibility between the developed formulation and human keratinocytes. This result was confirmed by evaluation of biomechanical skin properties including TEWL and SCH, which allow analysis of the integrity of the skin barrier after exposure to physical or chemical agents [30,48]. The results revealed an increase in TEWL and SCH values 5 min after topical application of AmB + BGEO gel; however, a tendency to return to the basal state was observed in both parameters after 25 min of assay, suggesting that AmB + BGEO gel does not cause irritation or damage to the skin surface of volunteers.

## 4. Materials and Methods

### 4.1. Materials

Amphotericin B (potency of 864 µg/mg) was purchased from Acofarma^®^ (Barcelona, Spain). *B. graveolens* essential oil was provided by the Unit Operations Laboratory of the Universidad Técnica Particular de Loja [25]. Dimethyl Sulfoxide (DMSO) was obtained from Alfa Aesar (Thermo Fisher, Karlsruhe, DE-BW, Germany). Glycerin, propylene glycol 400 and castor oil were supplied by Sigma-Aldrich (Madrid, Spain). Capric Triglyceride (Labrafac™ lipophile WL 1349), polyglyceryl-3 dioleate (Plurol^®^ oleique CC497), diethylene glycol monoethyl ether (Transcutol^®^ P), and propylene glycol monolaurate-type II (Lauroglycol™ 90) were obtained from Gatefossé (Saint-Priest, France). Carboxymethylcellulose (CMC), carbopol^®^ 940, sodium benzoate, parabens, and citric acid were obtained from Fagron Ibérica (Barcelona, Spain). HaCaT cell line was purchased from Cell Line Services (Eppelheim, DE-BW, Germany) and the reagents used for cell cultures were obtained from Gibco (Carcavelos, Lisbon, Portugal). The reagents for the MTT assay were obtained from Invitrogen Alfagene^®^ (Carcavelos, Lisbon, Portugal). A Millipore Milli-Q^®^ water purification system (Millipore Corporation; Burlington, MA, USA) was used. Finally, the chemicals and reagents were of analytical grade.

### 4.2. High-Performance Liquid Chromatography (HPLC)

AmB was quantified by a previously validated HPLC method [49]. The assay was carried out using a Waters HPLC with 2487 (UV/Vis) Detector & 717 Plus Autosampler (Waters, Milford, MA, USA). The mobile phase was a mixture of acetonitrile and glacial acetic acid 3.75% (65:35, *v*/*v*) filtered with a 0.45 µm PVDF membrane filter (Millipore Corp., Madrid, Spain). The assay was performed using a Kromasil C18 column (250 mm, 4.6 mm and 5 µm). The mobile phase was pumped at a flow rate of 0.5 mL/min and the injection volume was 10 µL. Finally, the elute was analyzed at 407 nm (wavelength of maximum absorbance of AmB).

### 4.3. Solubility Studies

The solubility of AmB in various solvents including DMSO, glycerin, propylene glycol 400, castor oil, Labrafac™ lipophile WL 1349, Plurol^®^ oleique CC497 and Lauroglycol™ 90 was evaluated using an excess of AmB and mixing by magnetic stirring for 30 min at 1500 rpm. The samples were equilibrated for 24 h and subsequently centrifuged at 9000 rpm for 10 min. The supernatant was extracted and diluted with methanol in order to quantify the dissolved AmB using a UV-Visible DR 6000 spectrophotometer (Hach^®^, Düsseldorf, DE-NW, Germany).

### 4.4. Formulation: Design and Analysis of 2^3^ Factorial Experiment

In this study, a 2^3^ factorial experiment was designed to examine the influence of three factors: the type of polymer (A), humectant (B) and preservative (C) at two levels, which are indicated by the signs “+” and “−” in Table 5 [50].

Eight different formulations were obtained with the 2^3^ factorial experiment using the ingredients at concentrations in accordance with the Handbook of Pharmaceutical Excipients and published data about efficacy and safety [51,52]. Furthermore, each formulation was composed of AmB (0.1%), BGEO (2%), DMSO (5%) and purified water. The pH was adjusted using citric acid (0.25%) in the CMC formulations and triethanolamine (1%) in the carbopol 940 formulations (Table 6).

In order to prepare the gels, polymers were hydrated for 30 min in water (mix 1). AmB was dissolved in DMSO under magnetic stirring for 10 min immediately followed by adding cosolvent and *B. graveolens* essential oil (mix 2). Citric acid and preservatives were dissolved in water (mix 3). Finally, mix 1 and 3 were mixed at 850 rpm using a IKA ULTRA-TURRAX T50 (Staufen, Germany) for 10 min, after which mix 2 was incorporated under the same stirring conditions. The final formulation was selected based on physical and chemical stability after 60 days of storage at 30 °C.

### 4.5. Characterization of AmB + BGEO Gel

The pH of AmB gel was determined using a pH meter GLP 22 (Crison Instruments, Barcelona, Spain).

Viscosity and rheological behavior of AmB gel were evaluated with a Haake Rheostress 1 rotational rheometer (Thermo Fisher Scientific, Kalsruhe, Germany) equipped with a cone–plate sensor system including a fixed bottom plate and a Haake C60/2Ti movable top cone (60 mm diameter, 2° angle, 0.105 mm gap). AmB gel was tested in duplicate 24 h after preparation. Viscosity values and flow curves were recorded during the ramp-up period from 0 to 50 s^−1^ (3 min), constant shear rate period of 50 s^−1^ (1 min), and a ramp-down period from 50 to 0 s^−1^ (3 min). Obtained data from the flow curve were fitted to different mathematical models including Newtonian, Bingham, Ostwald-de-Waele, Cross, Casson and Herschel–Bulkley. The model that best statistically describes the experimental data was selected according to the correlation coefficient value (r). Viscosity mean value (mPa·s) was determined at 50 s^−1^ from the constant shear rate period of viscosity curve. The determination of the disturbance of the microstructure during the test or “apparent thixotropy” (Pa/s) was evaluated by determining the area of the hysteresis loop.

The extensibility of AmB gel was analyzed in triplicate following the previously described method [53]. A sample of 1 g of formulation was placed on the center of the base plate of an extensometer. Afterwards, a glass plate (7.93 g) was placed on the sample without sliding and a series of weights (27.89, 57.82, 107.69, 157.55, 207.46, 237.50 and 307.69 g) were added at 1 min intervals. The extended area of the sample was recorded and then fitted to mathematical models using GraphPad Prism^®^ version 6.0 (GraphPad Software Inc., San Diego, CA, USA).

The drug content in the gel was determined by dissolving 100 mg of AmB gel in 5 mL of DMSO:methanol (1:1, *v*/*v*). After vortexing for 2 min, the solution was filtered and analyzed by HPLC method described in Section 4.2.

### 4.6. Stability Studies

After manufacturing, AmB + BGEO gel samples were stored at pre-established conditions of temperature and relative humidity (RH) in accordance with the Q1A(R2) ICH (International Conference on Harmonisation) Guidelines: 30 ± 2 °C/65 ± 5% RH and 40 ± 2 °C/75 ± 5% RH for 4 months [54]. Physical and chemical characterization of the formulation were carried out before and after storage in the aforementioned conditions using the method described in Section 4.5.

### 4.7. In Vitro Release Study

The release study of AmB from gel was performed using Franz diffusion cells of 13 mL and an effective diffusion area of 2.54 cm^2^ (FDC 400; Crown Grass, Somerville, NJ, USA). The receptor medium (RM) was a mixture of NN-dimethyl formamide, methanol and water (55:5:40, *v*/*v*). The conditions of the experiment were maintained at 32 °C and under continuous stirring in order to achieve sink conditions. A 0.45 µm nylon membrane was mounted between the donor and receptor compartments. A sample of 200 mg of AmB + BGEO gel was placed in the donor compartment and aliquots of 300 µL were collected from receptor compartment and replaced with the same volume of fresh RM at predetermined time intervals (2, 4, 6, 18, 21 and 24 h). The released amount of AmB (µg) from the formulation was determined by HPLC (Section 4.2) and plotted versus time (h) using GraphPad Prism^®^ 6.0 (GraphPad Software Inc., San Diego, CA, USA, 2014). The experiment was carried out in triplicate and data are represented as mean ± SD. Data from the release curve were fitted to several kinetic models including first order, Higuchi, Hyperbolic, Weibull and Korsmeyer–Peppa. Finally, the model with the highest coefficient of determination (r^2^) was selected.

### 4.8. Ex Vivo Permeation Study

The permeation studies were carried out using AmB + BGEO gel and AmB gel (*B. graveolens* essential oil-free AmB gel developed with the same qualitative and quantitative formula only without the incorporation of the essential oil) in order to compare and evaluate the role of the *B. graveolens* essential oil in drug permeation. These ex vivo permeation studies were carried out in Franz diffusion cells of 6 mL (0.64 cm^2^ diffusion area) using human skin obtained during an abdominal lipectomy of a healthy 38-year-old woman (Hospital of Barcelona, SCIAS, Barcelona, Spain). To that end, a written informed consent was provided by the volunteer in accordance with the Ethical Committee of the Hospital of Barcelona (number 001, dated 20 January 2016). To guarantee the integrity of the skin samples, Transepidermal water loss (TEWL) was measured using a Tewameter TM 300 (Courage & Khazaka Electronics GmbH; Cologne, Germany) and only those with results below 10 g/m^2^h were used. Skin samples (0.4 mm thick) were mounted between the donor and receptor compartments. Transcutol^®^ P was used as receptor medium (RM) which was kept at 32 °C and under stirring to guarantee sink conditions. A sample of 200 mg of AmB + BGEO gel or AmB gel was placed in the donor compartment in contact with the outer surface of skin. Aliquots of 300 µL were collected from the receptor compartment and replaced with the same volume of fresh RM at predetermined time intervals (2, 6, 18, 24, 28, 45 and 50 h). These aliquots were analyzed by HPLC (Section 4.2). Following permeation studies, these skin samples were removed from the Franz diffusion cells, washed with distilled water and cut along the edges in order to retain only the permeation area. Afterwards, skin samples were weighed and immersed in 1 mL of DMSO during 20 min under cold sonication using an ultrasonic bath in order to extract AmB retained in the skin (Qret, µg drug/g tissue/cm^2^). Finally, the resulting solution was filtered and analyzed by HPLC.

### 4.9. Efficacy Study: Antimicrobial Activity

The efficacy of the formulation was evaluated by determination of Minimum Inhibitory Concentration (MIC), which is defined as the lowest concentration of an antimicrobial agent necessary to inhibit the growth of a microorganism. The MIC was calculated by the broth microdilution method against three different strains of *Candida* spp. including *C. albicans* ATCC 10231, *C. glabrata* ATCC 66032 and *C. parapsilosis* ATCC 22019 (American Type Culture Collection, Manassas, VA, USA). This assay was carried out according to the guidelines outlined by the European Committee on Antimicrobial Susceptibility Testing (EUCAST) and the CLSI Reference Method M27-A3 [55,56].

A synthetic medium containing RPMI-1640, glutamine, pH indicator without bicarbonate and glucose 2% *w*/*v*: RPMI-1640 2% G (Invitrogen, Madrid, Spain) was used as culture medium. The yeast strains were first cultured on a Sabouraud Dextrose Agar Medium (Invitrogen, Madrid, Spain) at 30 °C for 48 h. The inoculums were prepared by suspending yeast colonies in sterile ¼ Ringer’s solution to achieve a density equivalent to 0.5 McFarland standards and counting in a Neubauer Chamber (1 to 5 × 10^6^ Colony Forming Unit, CFU/mL). Subsequently, a 1:10 dilution (1 to 5 × 10^5^ CFU/mL) was prepared to be used as the final inoculum.

A solution of AmB previously dissolved in DMSO (Free AmB) as well as samples of AmB + BGEO and AmB gel (*B. graveolens* essential oil-free AmB gel) were evaluated to perform a comparative analysis. The inoculum was used as a positive control and the culture medium as a negative control.

The experiment was performed using 96-well microdilution plates making serial double dilutions of the samples under analysis from 37.5 µg/mL to 0.0002 µg/mL for the formulations and 100% to 0.04% for the *B. graveolens* essential oil. Finally, 100 µL of the inoculum was added. Plates were read at t 0, 24 and 48 h after incubation at 30 °C with a microplate reader model 680 (Bio-Rad, Madrid, Spain) at 620 nm.

### 4.10. Tolerance Studies

#### 4.10.1. Cytotoxicity Studies by MTT Method

In vitro MTT cytotoxicy assay were performed using the human keratinocytes cell line HaCaT. Cells were adjusted at 2 × 10^5^ cell/mL, seeded in 96-well plate for 24 h in Dubelcco’s Modified Eagle’s Medium (DMEM) with high glucose content buffered with 25 mM HEPES, and supplemented with 1% non-essential amino acids, 100 U/mL penicillin, 100 g/mL streptomycin and 10% heat inactivated Fetal Bovine Serum (FBS). Next, the cells were incubated with different dilutions of the AmB + BGEO gel ranging from 1/50 to 1/2000 for 24 h. Later, the HaCaT cells were washed with 1% sterile PBS and incubated with MTT (Sigma-Aldrich Chemical Co, St. Louis, MO, USA) solution (2.5 mg/mL) for 2 h at 37 °C. The medium was then carefully removed and 0.1 mL of solubilization reagent (99% DMSO) was added to lyse the cells and dissolve the purple MTT crystals. Cell viability was measured at 570 nm in a microplate photometer Varioskan TM LUX (Thermo Scientific, Waltham, MA, USA). A negative control was processed in parallel for comparison. The results were expressed as percentage of cell survival relative to the control (untreated HaCaT cells; 100% viability) using the following equation:

(2) $$\%\;\mathrm{Cell}\;\mathrm{viability}=\frac{\mathrm{Abs}\;\mathrm{sample}}{\mathrm{Abs}\;\mathrm{control}}\times 100$$

#### 4.10.2. In Vivo Tolerance

Measurements of transepidermal water loss (TEWL) and stratum corneum hydration (SCH) of the ventral area of the forearm of 12 volunteers (6 men and 6 women; ages between 20 and 35 years) with healthy skin were performed with prior written informed consent. This study was approved by the Ethics Committee of the University of Barcelona (IRB00003099) in accordance with the principles from the Declaration of Helsinki.

The time intervals of each measurement were 5, 15 and 25 min after absorption of AmB + BGEO gel in the application area. Readings were recorded using a Tewameter^®^ TM300 and Corneometer^®^ 825 (Courage & Khazaka Electronics GmbH, Cologne, Germany) for TEWL and SCH, respectively. For TEWL measurements, the probe was pressed and held on the skin for 2 min and the results are expressed as g/cm^2^/h. For the SCH values, the probe was pressed on the skin to measure the dielectric constant of the skin where measurements were given in arbitrary units (AU). The results of TEWL and SCH were recorded as the median, minimum and maximum (*n* = 12).

## 5. Conclusions

In conclusion, the present study provides evidence that the topical gel of AmB enriched with *B. graveolens* essential oil offers appropriate characteristics for skin application including biocompatible values of pH, pseudoplastic behavior, optimal spreadability and acceptable tolerability, all of which make it an appealing and suitable formulation for human use. Furthermore, the incorporation of *B. graveolens* essential oil improved the biopharmaceutical profile of the drug, facilitating its penetration through the SC and its retention into the skin and consequently promoting a local effect in the target area linked to enhanced antifungal activity against *C.albicans*, *C. glabrata* and *C. parapsilosis.* Therefore, this formulation could constitute a promising alternative in the treatment of cutaneous candidiasis which encourages further research to explore its use in clinical practice.

## Figures and Tables

**Table 1:** Physical and chemical characterization of the 8 formulations obtained by 2^3^ factorial experiment after one and 60 days of preparation and storage at 30 °C.

1 Day				60 Days		
Formulation	Appearance	pH	Drug Content (%)	Appearance	pH	Drug Content (%)
F1	Homogeneous	4.80 ± 0.03	99.68 ± 0.16	Precipitates	4.12 ± 0.09	96.78 ± 1.67
F2	Homogeneous	6.76 ± 0.05	99.73 ± 0.12	Homogeneous	4.98 ± 0.07	98.25 ± 0.82
F3	Homogeneous	5.85 ± 0.09	99.65 ± 0.08	Homogeneous	5.57 ± 0.04	99.18 ± 0.43
F4	Homogeneous	6.81 ± 0.07	99.66 ± 0.10	Lumps	6.02 ± 0.10	98.86 ± 1.35
F5	Homogeneous	5.28 ± 0.01	99.52 ± 0.09	Homogeneous	4.16 ± 0.15	98.16 ± 0.98
F6	Homogeneous	7.12 ± 0.08	99.59 ± 0.13	Precipitates	6.15 ± 0.09	97.63 ± 1.82
F7	Homogeneous	5.92 ± 0.04	99.81 ± 0.09	Homogeneous	5.05 ± 0.07	98.83 ± 0.66
F8	Homogeneous	6.70 ± 0.06	99.65 ± 0.11	Precipitates	5.72 ± 0.25	97.15 ± 1.68

**Table 2:** Final formulation of AmB + BGEO gel.

Component (%)	
Amphotericin B (AmB)	0.1
*B. graveolens* essential oil (BGEO)	2
Carboxymethylcelullose (CMC)	3
Propylene glycol 400	20
Sodium benzoate	0.02
Citric acid	0.2
Dimethylsulfoxyde (DMSO)	5
Water	69.68

**Table 3:** Stability studies of AmB + BGEO gel.

Time (Days)	30 ± 2 °C/65 ± 5% RH			40 ± 2 °C/75 ± 5% RH		
	Appearance	pH	Drug Content (%)	Appearance	pH	Drug Content (%)
1	Homogeneous	5.85 ± 0.09	99.65 ± 0.08	Homogeneous	5.83 ± 0.07	99.67 ± 0.12
60	Homogeneous	5.57 ± 0.04	99.18 ± 0.43	Homogeneous	5.43 ± 0.15	98.91 ± 0.17
120 days	Homogeneous	5.48 ± 0.12	98.79 ± 0.66	Homogeneous	5.12 ± 0.07	98.56 ± 0.32

**Table 4:** MIC against different cultures of *Candida* spp., Free AmB, AmB gel, AmB + BGEO gel and *B. graveolens* essential oil (BGEO) after incubation at 30 °C for 48 h (*n* = 3).

Tested Species	Origin	MIC (µg/mL)			% (*v*/*v*)
		Free AmB	AmB Gel	AmB + BGEO Gel	BGEO
*C. albicans*	ATCC 10231	0.15	0.58	0.29	12.50
*C. grabrata*	ATCC 66032	0.60	0.58	0.39	6.25
*C. parapsilosis*	ATCC 22019	0.30	1	0.58	12.50

**Table 5:** Factors studied in the factorial experimental design and their levels.

Factors	Levels	
	(+)	(−)
A: Polymer	Carboxymethylcellulose	Carbopol
B: Cosolvent	Glycerin	Propylene glycol 400
C: Preservative	Sodium benzoate	Parabens

**Table 6:** Formulations developed from the 2^3^ factorial experiment.

Component	%	F1	F2	F3	F4	F5	F6	F7	F8
CMC	3	*		*		*		*	
Carbopol 940	1		*		*		*		*
Glycerin	20	*	*			*	*		
Propylene glycol 400	20			*	*			*	*
Sodium benzoate	0.02			*		*	*		*
Parabens	0.02	*	*		*			*	
Citric acid	0.20	*		*		*		*	
Triethanolamine	1		*		*		*		*
AmB	0.1	*	*	*	*	*	*	*	*
DMSO	5	*	*	*	*	*	*	*	*
BGEO	2	*	*	*	*	*	*	*	*
Water	sq	*	*	*	*	*	*	*	*

*BGEO* = *Bursera graveolens* essential oil, CMC = Carboxymethylcelullose, AmB = Amphotericin B, DMSO = Dimethyl Sulfoxide, sq = Sufficient quantity, * presence of the component (featured in the left-most column).

## Data Availability

Data is contained within the article.
